# Editorial: Case reports in structural interventional cardiology: 2024

**DOI:** 10.3389/fcvm.2025.1681790

**Published:** 2025-09-05

**Authors:** Sunny Goel

**Affiliations:** ^1^ Department of Cardiology, Mount Sinai South Nassau, Oceanside, NY, United States; ^2^ Department of Cardiology, Mount Sinai Hospital, New York, NY, United States

**Keywords:** TAVI, teer, CRT, prosthetic valve, structural cardiology

**Editorial on the Research Topic**
Case reports in structural interventional cardiology: 2024

As I reflect on the remarkable Research Topic of structural interventional cardiology case reports published in 2024, I am struck by a singular truth: the evolution of our field has never been more intricately tied to individual patient stories. These case reports, often representing rare or first-in-field experiences, highlight not just what is technically possible but what is clinically necessary when textbook scenarios fall short. As a structural interventionalist, I see in these reports a mirror of the daily challenges we face—uncertainty, interdisciplinary collaboration, technical creativity, and, ultimately, courage.

The 2024 compilation in Case Reports in Structural Interventional Cardiology offers a spectrum of such experiences, and each case resonates deeply. For instance, the report by Bi et al. describes a transcatheter endovascular aortic repair (TEVAR) in a pregnant woman with acute type B aortic dissection and underlying Takayasu's arteritis, performed immediately following cesarean section (Bi et al.). This case encapsulates the kind of high-stake, high-coordination decision-making that defines modern structural intervention. Pregnancy, autoimmune vasculopathy, dissection, and urgent peripartum management are each daunting alone—together, they form a procedural Gordian knot. Yet through collaboration and ingenuity, the team achieved an outcome that would have been inconceivable just a decade ago.

On the opposite end of the age spectrum, Pervunina et al. present a poignant and technically demanding case of transcatheter aortic valve implantation (TAVI) in a 13-year-old with Singleton–Merten syndrome and critical aortic stenosis (Pervunina et al.). This rare genetic condition is associated with severe calcifications and dental anomalies and is almost never encountered in interventional practice. The thoughtful pre-procedural planning and precise valve selection in this case underscore the importance of a pediatric structural heart mindset—one that acknowledges syndromic nuances while pushing the boundaries of adult-derived technologies.

Innovation in preprocedural modeling is exemplified by Zhang et al., who tackled a quadricuspid aortic valve using patient-specific three-dimensional (3D) printing and computational fluid dynamics (CFD) to guide transcatheter therapy (Zhang et al.). This case is more than a technical report; it is a glimpse into the future. As an operator, I have witnessed how adjunctive technologies like CFD modeling can help predict gradients, leaflet motion, and paravalvular leak. In unusual valve morphologies such as quadricuspid anatomy, these tools can mean the difference between a marginal and an optimal outcome.

Other reports focus on unexpected physiological responses to seemingly routine interventions. Pang et al. describes the development of Takotsubo syndrome following mitral transcatheter edge-to-edge repair (TEER), an emotional reminder that the structural lab is not immune to the systemic and psychological interplay of heart and mind (Pang et al.). The transient left ventricular dysfunction post-TEER raises important questions: Do procedural stressors, sedation regimens, or intra-atrial manipulation increase susceptibility in vulnerable patients? Are we overlooking subtle cues of pre-existing catecholaminergic stress? This case humbles us to consider not only valves and annuli, but the patient as a whole.

Hou et al. report an elegant percutaneous solution to close an artificial vascular anastomotic fistula following prior aortic replacement (Hou et al.). In this case, surgical re-intervention posed significant risk, and yet the percutaneous route was approached with creativity, leveraging tools outside their original intended use. Such cases are common in the structural arena, where interventionalists must constantly think beyond devices and labels to meet clinical needs.

Complications following prior structural procedures also feature prominently. A case by Zhou et al. discusses valve thrombosis post valve-in-valve TAVI in a patient with prior prosthetic valve endocarditis (Zhou et al.). This report is a sobering look at the complexity of managing layered pathologies—infection, degeneration, thrombosis—across bioprosthetic and transcatheter interfaces. It emphasizes the critical importance of patient selection, long-term anticoagulation strategies, and the need for vigilant follow-up imaging.

Finally, the report by Özmen et al. on the challenges of extracting defective CRT-D ventricular lead reminds us that structural interventionalists often work in close proximity to electrophysiology domains (Özmen et al.). Although not a structural heart case in the classical sense, this case shares our procedural terrain—dense fibrosis, device entanglement, and risk of catastrophic vascular or myocardial injury. As our specialties continue to overlap, we must remain agile, collaborative, and prepared to navigate shared complications.

Personally, I find these case reports invigorating. Each one takes me back to similar moments in my practice—the gut feeling that something atypical is brewing, the 2 a.m. phone call from a colleague about an anatomy we have never encountered, the excitement and trepidation of trying something “off-label” because it's the only option left. These stories remind us why structural cardiology is both exhilarating and humbling.

Beyond their clinical and technical contributions, case reports serve as vital educational tools. For fellows-in-training, early-career interventionalists, and even seasoned operators, these reports distill months of decision-making into digestible, reflective narratives. They humanize the field, placing patient stories above procedural metrics. In an era of increasingly algorithm-driven care, case reports preserve the art of medicine.

In closing, I extend my sincere thanks to all the authors, reviewers, and editorial team members who brought this 2024 collection to life. Through these contributions, we continue to challenge our limits, expand our collective wisdom, and above all, improve care for the patients who trust us with their most vulnerable moments.

